# Coagulopathy in Acute Promyelocytic Leukemia: Can We Go Beyond Supportive Care?

**DOI:** 10.3389/fmed.2021.722614

**Published:** 2021-08-17

**Authors:** Bryan C. Hambley, Ciprian Tomuleasa, Gabriel Ghiaur

**Affiliations:** ^1^Division of Hematology/Oncology, Department of Internal Medicine, University of Cincinnati, Cincinnati, OH, United States; ^2^Department of Hematology, Iuliu Hatieganu University of Medicine and Pharmacy, Cluj Napoca, Romania; ^3^Department of Hematology, Ion Chiricuta Clinical Cancer Center, Cluj Napoca, Romania; ^4^Medfuture Research Center for Advanced Medicine, Iuliu Hatieganu University of Medicine and Pharmacy, Cluj Napoca, Romania; ^5^Department of Oncology, Sidney Kimmel Comprehensive Cancer Center, Johns Hopkins University School of Medicine, Baltimore, MD, United States

**Keywords:** APL, ATRA, hyperfibrinolysis, hemorrhage, thrombosis, delayed bleeding

## Abstract

Acute promyelocytic leukemia (APL) is characterized by frequent complications due to a distinct coagulopathy. While advances in treatments have improved long-term survival, hemorrhagic and thrombotic complications remain the most common causes of death and morbidity. Improved understanding of the mechanisms of the coagulopathy associated with APL may lead to therapeutic interventions to mitigate the risk of hemorrhage and thrombosis.

## Introduction

Acute promyelocytic leukemia (APL) is caused by a translocation of the retinoic acid receptor alpha (*RAR*α) on chromosome 17, most commonly with the promyelocytic leukemia gene (*PML*) on chromosome 15, which leads to clonal proliferation of promyeloblasts (1). The specific focus of this review is APL with *PML*-*RAR*α, classified by the 2016 World Health Organization (WHO) criteria as a distinct entity apart from rare variants of promyelocytic leukemia (2).

Long-term survival outcomes for APL are now higher than any other acute leukemia as a result of advances such as all-trans retinoic acid (ATRA) and arsenic trioxide (ATO) (3, 4). For APL patients who survive the first 30 days, over 90% are cured of the disease (4, 5). The increasing use of combined ATRA and ATO for patients with low and intermediate-risk APL has improved long-term cure rates in the disease (6). Nevertheless, death within the first 30 days after diagnosis remains the most common cause of treatment failure (7). Indeed, an updated analysis from the Swedish Acute Leukemia Registry revealed a 25% mortality rate in the first 30 days of therapy, with no improvement from 1997–2008 compared with 2009–2013 (8). Hemorrhage, particularly intracranial hemorrhage, is the most common cause of early death (7–9). The highest risk period for early death and hemorrhagic complications is in the first 4 days of therapy, though almost 50% of early deaths and hemorrhagic complications occur between day 5 and 30 (8, 10–12). Additionally, venous and arterial thrombosis occurs in up to 20% of patients with APL (13–15). Common thrombotic events include deep vein thrombosis, pulmonary embolism, myocardial infarction, and ischemic cerebrovascular events (13–15). The increased risk of both hemorrhagic and thrombotic complications in APL highlights the unique mechanisms that govern the coagulopathy of these patients. Recent work allows us to better understand how current anti-leukemia treatments impact the coagulopathy associated with APL.

**Figure 1:**
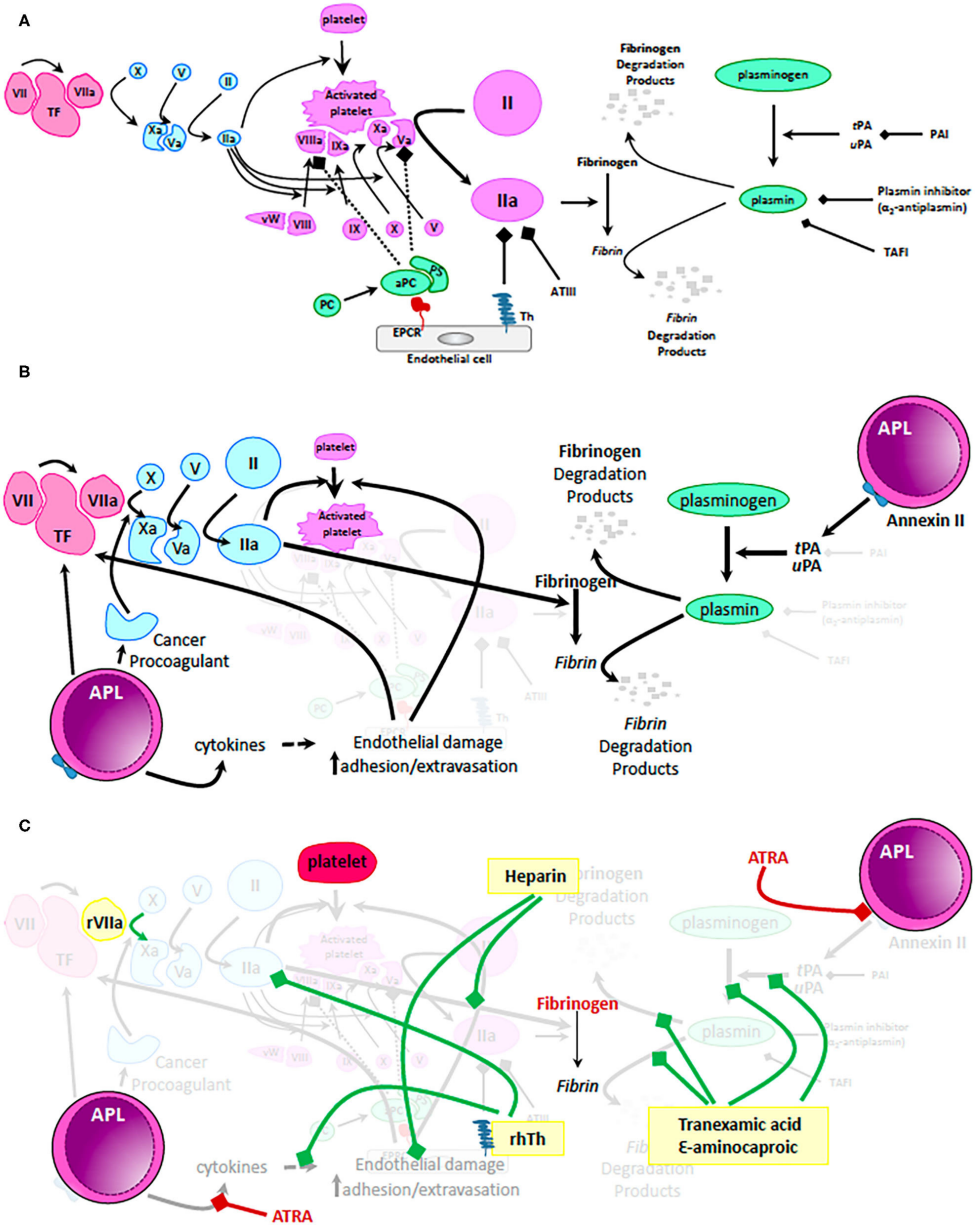
Mechanisms of coagulopathy in APL. **(A)** Physiologic mechanisms of coagulation and anticoagulation. Tissue factor (TF) released by trauma to the vascular wall activates factor VII. Small amounts of activated factor VII (VIIa) activates factor X which in turn activates factor V. Activated factor X (Xa) together with activated factor V (Va) cleaves and activates prothrombin to thrombin (IIa). Only small amounts of thrombin are generally produced via this mechanism. These levels are insufficient to form a robust fibrin clot but can activate platelets, factor VIII, factor X, and factor V resulting in generation of clinically significant amounts of IIa on the surface of activated platelets. High levels of IIa are now sufficient to cleave fibrinogen to fibrin and thus, stabilize the clot. During normal hemostasis, clot formation is restricted to the place of endothelial damage via: (i) mechanisms dependent on endothelial cell expression of thrombomodulin (Th - binds and inactivates IIa) and endothelial protein C receptor (EPCR – binds and activates protein C, which together with protein S inactivates IIa, Va, and VIIIa); (ii) antithrombin III (ATIII – binds and inactivates IIa) and (iii) fibrinolysis pathway that results in generation of plasmin from plasminogen. tPA, tissue plasminogen activator; uPA, urine plasminogen activator; PAI, plasminogen activator inhibitor; TAFI, thrombin activatable fibrinolysis inhibitor. **(B)** Mechanisms by which the coagulation pathways are dysregulated in patients with APL. Malignant promyelocytes produce excessive amounts of TF, cytokines (i.e., IL1β, TNFα) and cancer procoagulant leading to unrestricted generation of massive amounts of IIa and consumptive coagulopathy. In addition, APL promyeloblasts express high levels of Annexin II, a receptor and activator of tPA/uPA resulting in uncontrolled generation of plasmin and hyperfibrinolysis. **(C)** Mechanistic impact of therapeutic interventions routinely used in APL. Standard of care recommendations are depicted with red highlighting, text and bars (platelets, fibrinogen, or fresh frozen plasma and ATRA). Of note, ATRA can rapidly (within minutes) decrease expression of Annexin II and thus, ameliorate hyperfibrinolysis. In yellow boxes are proposed interventions that are not currently recommended as routine but that may positively influence the coagulopathy of some patients with APL. Green bars depict suggested mechanisms of action of these proposed interventions. Recombinant VIIa has been used in patients with life threatening intracranial bleeding; low dose heparin not only decreases consumption of coagulation factors by blocking IIa but also alleviates endothelial damage; fibrinolysis inhibitors (tranexamic acid and ε-aminocaproic acid) can mitigate hyperfibrinolysis and excessive fibrinogen consumption. Most exciting is the potential use of recombinant human thrombomodulin (rhTh) to not only balance IIa activity but also palliate some of the cytokine dependent endothelial damage. While these interventions may have a role to play in management of coagulopathy in APL, further clinical trial data are needed before making firm recommendations.

At steady state, blood flow is maintained in the face of vascular injury via complex interactions that balance primary and secondary hemostasis (mitigating blood loss) with antithrombotic mechanisms (preventing obstruction of flow and loss of coagulation factors) (Figure 1A). Our understanding of the molecular processes that govern hemostasis and thrombosis have significantly improved during the last three decades. To this end, platelets play a central role not only during primary hemostasis but also in the cellular model of secondary hemostasis (16). More so, it is now clear tissue-specific differences in basal fibrinolytic capacity and endothelial cell expression of thrombomodulin (TM) and endothelial protein C receptor (EPCR) contribute to different patterns of bleeding and thrombosis (17). Lastly, the past 30 years have seen rapid development and FDA approval of a number of drugs meant to mitigate uncontrolled bleeding (i.e., recombinant FVIIa, tranexamic acid, and ε-aminocaproic acid) and clotting (direct thrombin inhibitors, direct factor Xa inhibitors) (18–20). Improved access to supportive measures (replacement of platelets and coagulation factors) together with better understanding of the molecular mechanisms of action of unfractionated and low molecular weight heparins provide a vast armamentarium to approach patients with impaired hemostasis and thrombosis. Yet, progress in the management of these complications in patients with APL lags behind. This lag in therapeutic advancement may be related to difficulties in studying a rare disease, the need for therapeutic interventions to begin very early to mitigate the early complication rate, and lack of commercial support for clinical trials of approaches that utilize blood products or generic medications.

This review highlights the unique pathophysiology of hemostasis and thrombosis in APL and the major clinical reports on the frequency and nature of hemorrhagic and thrombotic events. We will emphasize the commonly used interventions in the management of APL with an eye toward their potential impact on the mechanisms responsible for the coagulopathy present in this disease. Lastly, this review will not only present the current interventions used to mitigate the hemorrhagic risks but also emphasize potential treatment strategies and novel agents that merit clinical investigation to decrease coagulopathy-related death in these patients.

## Bleeding Diathesis in APL

### Mechanisms of Hemorrhage in APL

Patients with APL have a distinctively low fibrinogen and increased fibrinogen degradation products and fibrin degradation products. In fact, fibrinogen levels <150 mg/dL in a patient with acute myeloid leukemia and relatively low white blood cell count raise the clinical suspicion for APL (21). Even though secondary fibrinolysis contributes to the coagulopathy seen in patients with APL, primary hyperfibrinolysis dominates the pathophysiology and drives the increased risk of major bleeding in these patients (22–24). Primary hyperfibrinolysis in APL relies on tissue plasminogen activator (tPA), urokinase plasminogen activator (uPA), and annexin II, while secondary hyperfibrinolysis relies on systemic breakdown of clots and microvascular clotting/lysis typically seen in disseminated intravascular coagulopathy (22, 23). Promyeloblasts may interact with fibrinolysis at multiple points of the pathway (Figure 1B) but of particular interest is their expression of annexin II, the receptor for uPA and tPA (23, 25). High levels of annexin II on APL cells contribute to abnormal activation of plasmin and subsequent degradation of fibrin and fibrinogen, resulting in critically low levels of this product (23). Once released, plasmin binds, inactivates, and is inactivated by α_2_-antiplasmin-an inhibitor of the fibrinolytic pathway (23, 26). In addition to high expression of annexin II on APL cells, serum from these patients has reduced levels of α_2_-antiplasmin, contributing to further uncontrolled fibrinolysis (25). Recent studies have called into question the role of annexin II in hyperfibrinolysis seen in APL and reported low circulating levels of this protein. In these studies, it was the increased urokinase-type plasminogen activator receptor (uPAR) that claimed a role in fibrin and fibrinogen degradation (27). Since cellular annexin II levels were not assessed in these studies, the contribution of this mechanism cannot be refuted. Nevertheless, further studies may clarify the differential contribution of uPAR and annexin II mechanisms to the hyperfibrinolysis present in patients with APL.

In addition to the annexin II-plasmin axis, other pathways triggered by presence of abnormal myeloid cells could set the stage for the dramatic drop in fibrinogen seen in APL. Abnormal elastase activity and activation of matrix metallopeptidases may contribute to the initial bleeding risk of patients with APL, though more research is needed to define their role (28–31). In addition, a number of investigators have reported increased levels of tissue factor pathway inhibitor (TFPI) in patients with APL. Further studies are necessary to define not only the pathophysiologic role of TFPI in the hemorrhagic events seen in APL but also the therapeutic potential of recombinant human TFPI in these patients (32).

Beyond systemic markers of hyperfibrinolysis and coagulopathy, local cell-based factors may play a role in the distribution of hemorrhagic events in APL. The increased expression of annexin II on central nervous system endothelial cells may contribute to the high incidence of intracerebral hemorrhage in APL (33). Thrombocytopenia is a common finding in APL. Nevertheless, the degree to which thrombocytopenia leads to hemorrhagic events remains unclear (11, 34). Coagulopathy in APL shares some characteristics with DIC in sepsis, but the quantitative and qualitative platelet defects in APL are more profound (35). While antithrombin III levels are reduced in DIC, they are usually preserved in APL (36). The combination of decreased capacity to activate platelets and lower levels of platelet concentration does provide further physiologic rationale for the hemorrhagic tendency in APL.

### Clinical Bleeding

Hemorrhagic events in APL follow a characteristic clinical pattern. The most frequent cause of fatal bleeding is intracranial hemorrhage (8, 37, 38). Among 22 early hemorrhagic deaths in the Swedish Acute Leukemia Registry between 1997 and 2013, 21 were due to intracranial hemorrhage (8). A report from the PETHEMA Group of patients treated on clinical trials with ATRA and idarubicin found 37 of 66 deaths during induction were related to hemorrhage, 24 intracranial and 12 intrapulmonary (39). In this report, while most hemorrhagic deaths occurred in the first 10 days, fatal bleeding events continued until day 23 of induction (39). Table 1A details clinical reports of hemorrhage and associated risk factors.

**Table 1:** Incidence of fatal hemorrhage and risk factors in published reports.

**References**	**Years**	**Incidence of early death**	**Incidence of early hemorrhagic death**	**Risk factors for early death**
Lehmann et al. (8)	1997–2013	49/195 (25.1%)	22/195 (11.3%)	Age >55 yearsWorse performance statusWBC >5 ×10^9^/L
Xu et al. (9)	2003–2013	49/212 (23.1%)	37/212 (17.5%)^a^	ECOG PS 3–4High-Risk SanzIncreased creatinineIncreased LDH
Park et al. (7)	1992–2007	242/1,400 (17.3%)	N/A	Age >55 years
Mantha et al. (11)	1992–2010	N/A	37/995 (3.7%)^b^	WBC ≥ 20 ×10^9^/L^3^ECOG PS 3–4^c^
Asou et al. (40) and Yanada et al. (12)	1997–2002	13/283 (4.6%)^b^	9/283 (3.2%)^b^	Fibrinogen <1.0 g/LWBC ≥ 20 ×10^9^/L^3^ECOG PS 2–3

a *Includes all early deaths associated with bleeding, including causes of death where bleeding and other events such as leukostasis were deemed combined causes of death*.

b *Clinical trial population rather than registry/center database of all patients with APL*.

c *Risk factors for hemorrhagic death rather than all early death*.

One extensive report of intracranial hemorrhage in 12 patients with APL and 39 with other AML showed various locations of bleeding (intraparenchymal, subarachnoid, subdural, epidural, and intraventricular) (37). Most of the intracranial hemorrhages in this study, except those with subdural hemorrhage, resulted in death (37).

Higher white blood cell (WBC) count and decreased fibrinogen at presentation is associated with increased risk of severe or fatal bleeding (12, 39, 41–43). Notably, the association between fibrinogen level at diagnosis and increased hemorrhagic events was not found in other retrospective studies (39, 44). Even though thrombocytopenia is common in APL, reports vary on the association of baseline thrombocytopenia with increased hemorrhage (39, 42–44). More so, prothrombin time (PT) and activated partial thromboplastin time (aPTT) are not consistently associated with bleeding risk (11, 45). Thus, standard coagulation and laboratory assessments of patients with APL do not provide a complete understanding of the risk of hemorrhage in these patients. We and others have used specialized tests that capture a more comprehensive picture of coagulopathy such as thromboelastography (TEG) in order to better understand the coagulopathy in certain patients with APL as well as other types of leukemia (46). Prolonged R time and decreased angle and maximum amplitude have been observed amongst patients with clinical bleeding (46). The interpretation of these tests in the context of APL remains investigational and their restricted availability outside of large academic centers makes them impractical for most patients with this disease.

In addition to typical coagulopathy assessments, other factors are associated with increased incidence of hemorrhagic events in APL. The PETHEMA Group report found a strong association on multivariate analysis of abnormal renal function and fatal hemorrhage, a metric not universally analyzed in other reports (39). While one could hypothesize that abnormal renal function may result in impaired platelet activity, this causative relation has not been formally proven. Similarly, impaired functional status, as measured by the Eastern Cooperative Oncology Group Performance Status, is associated with increased risk of hemorrhage or early death (11, 47). The mechanism responsible for increased hemorrhage or early death in patients with poor performance status is unknown, limiting the ability to adjust therapy to mitigate this risk.

More so, while the first 7 days of therapy are associated with the highest incidence of bleeding events and early death, hemorrhagic risk and fatal bleeding events persist through the first month of therapy (10, 11, 42). Thus, bleeding complications during induction but past the first few days from diagnosis may have divergent pathophysiology and depend more on treatment response and fibrinogen consumption, and less on factors such as WBC at diagnosis (9, 10, 42).

## Hypercoagulability in APL

### Mechanisms of Thrombosis in APL

Malignant cells in APL, like those from solid cancers, induce a procoagulant state. This thrombotic tendency is mostly mediated by expression of cancer procoagulant and tissue factor, though additional mechanisms are also involved. For instance, microparticles generated by various cells are elevated in the plasma of patients with APL compared with normal individuals (48, 49). These microparticles express tissue factor and induce widespread thrombin generation (48–50). Compared with other acute leukemias, levels of cancer procoagulant are more elevated in APL, leading to activation of factor X and increased propensity for thrombosis (51, 52).

In addition, APL promyeloblasts overexpress various cytokines including interleukin-1β (IL-1β), interleukin-6 (IL-6), and tumor necrosis factor α (TNF-α) (50). IL-1β and TNF-α augment the activity of tissue factor and plasminogen activator inhibitor-1 (PAI-1) and may contribute to hypercoagulability (53, 54). TNF-α downregulates transcription of thrombomodulin, with an *in vivo* study demonstrating >80% reduction in thrombomodulin levels after treatment with TNF-α (55, 56). Thus, low levels of thrombomodulin are thought to play a central role in the coagulopathy associated with APL. Initial clinical trials testing the use of recombinant human soluble thrombomodulin (rTM) to mitigate the hypercoagulability of APL have been published (57, 58). The use of rTM for patients with APL has become common in Japan, though a recent retrospective report did not find evidence of improved clinical outcomes (59).

#### Clinical Thrombosis

While bleeding is the most feared complication of APL, thrombosis is a common but under-recognized presentation. Multiple reports reveal a 5–20% risk of thrombotic events during induction therapy for APL, with the largest PETHEMA report finding 39/759 (5.1%) with a thrombotic event (15, 60–63). The most common locations of thrombosis are cerebral infarction, deep vein thrombosis, pulmonary embolism, and acute myocardial infarction (60–63). Table 1B details clinical reports of thrombotic complications and their incidence in patients with APL. Small sample sizes of the available retrospective reports have hampered the efforts to define risk factors for thrombosis in APL. Interventional research to guide management of thrombosis, particularly life-threatening thrombosis such as cerebral infarction, is currently lacking. A 2019 European LeukemiaNet (ELN) panel recommended the consideration of heparin with dose modifications based upon degree of thrombocytopenia for severe thrombosis (64). The approach to thrombosis remains a difficult decision for the bedside clinical team.

**Table 1B:** Incidence of thrombosis and risk factors in published reports.

**References**	**Years**	**Incidence of thrombosis**	**Location of thrombosis**
Breccia et al. (13)	1993–2001	11/124 (8.9%)	DVT (5) Cardiac (4) Intracranial (2)
Rashidi et al. (14)		N/A^a^	DVT (27/94) Cardiac (25/94) Intracranial (27/94)
Mitrovic et al. (15)	2004–2010	13/63 (20.6%)	DVT (7) Cardiac (2) Intracranial (2) Budd-Chiari (1) Retinal vein (1)
Montesinos et al. (60)	1996–2005	39/759 (5.1%)	DVT (17) PE (5) Cardiac (4) Intracranial (10)
Bai et al. (63)	2013–2018	6/33 (18.2%)^b^	DVT (2) Intracranial (4)

a *Literature review*.

b *Only included patients with WBC at diagnosis >10 × 10^9^/L*.

## Impact of Therapy On Coagulopathy

Reports of the ongoing risk of thrombosis and hemorrhage during the initial 30 days of treatment for APL underscore the pathophysiologic importance of the interaction of therapies with the promyeloblasts and resulting alterations in coagulopathy (Figure 1C).

Promyeloblasts secrete high amounts of proteases which degrade collagen and thereby activate enzymes of the coagulation cascade (65). Conventional chemotherapy, including anthracyclines, may lead to a flare-up of coagulopathy and later increased risk of developing a hemorrhagic syndrome. Studies of anthracycline-free regimens using arsenic trioxide (ATO) showed less early death and severe thrombocytopenia, with fewer reported cases of hemorrhagic death (4, 66). One report of two patients treated with gemtuzumab ozogamicin (GO) identified marked increase in fibrinogen degradation products after initial dose, however the impact of GO on coagulopathy in APL remains understudied (67).

ATRA's impact on coagulopathy in APL is more thoroughly studied and better understood compared with other therapies. ATRA-based regimens lead to less expression of tissue factor and annexin II by APL promyeloblasts, with a subsequent decrease of the dynamic coagulopathy after treatment initiation (68, 69). ATRA leads to a downregulation of tissue factor expression and cancer procoagulant activity in APL (51, 70, 71). A study using NB4 APL cells that were either sensitive or resistant to ATRA-induced differentiation revealed that ATRA decreases the activity of cancer procoagulant as the blasts differentiate (51). In contrast, ATRA downregulates tissue factor activity independent of pro-differentiation effects (51). More so, ATRA-exposed NB4 cells demonstrate increased thrombomodulin expression while annexin II levels are reduced within 24 h of initiation of ATRA treatment (23, 69, 70). In fact, even though studies evaluating clinical outcomes stratified by time to ATRA initiation are limited by their retrospective nature and small sample size, they confirm the pathophysiologic rationale for rapid treatment initiation and have led most centers to expedite the initiation of treatment with ATRA at first suspicion of APL (64).

Nevertheless, the effects of ATRA on the coagulopathy of APL are complex and not fully elucidated. For instance, by inducing differentiation of malignant promyelocytes, ATRA could enhance ETosis in APL. This newly recognized form of cell death allows the nuclear chromatin to come into direct contact with intracellular enzymes and be released outside the cells (72). Outside the cells, chromatin is organized into NETs (neutrophil extracellular traps) physiologically designed to catch bacteria; however, in patients with APL this may cause endothelial damage and not only further activate the coagulation cascade but also trigger intracranial bleeding, alveolar hemorrhage and differentiation syndrome (73–75). The hypothesized ATRA-induced ETosis may contribute to the delayed bleeding found in patients with APL though further research is necessary to establish to what extent this mechanism is clinically significant.

## Practical Strategies to Mitigate Coagulopathy in APL

### Transfusion

Standard supportive care to mitigate the hemorrhagic complications of APL includes platelet transfusion to goal >30–50 × 10^9^/L, cryoprecipitate to maintain fibrinogen >100–150 mg/dL, and fresh frozen plasma to maintain INR <1.5 (64). Limited prospective data exist to define optimal transfusion thresholds. Guidelines and clinical practice have grown to follow those used in the protocols for major trials in APL, such as platelet transfusion to maintain >30 × 10^9^/L and plasma to maintain fibrinogen >150 mg/dl in the 2013 ATRA/ATO trial published by Lo-Coco et al. (4).

### Antifibrinolytics

Given the predominant role of hyperfibrinolysis in APL, antifibrinolytics were evaluated to decrease bleeding risk. A retrospective study of 268 patients in the pre-ATRA era showed no significant difference in bleeding between those treated with antifibrinolytics or supportive care alone (76). Additionally, the PETHEMA group's LPA96 and LPA99 trials had similar rates of early death (5.1 and 5.0%, respectively) despite the use of prophylactic tranexamic acid in LPA99 and not LPA96 (39). More so, in these studies, there was an increased risk of thrombosis associated with the use of tranexamic acid without any reduction in hemorrhagic events (77). The use of antifibrinolytics in specific clinical situations, such as in patients with hyperfibrinolytic coagulation profiles or actively bleeding patients, has not been systematically studied. Thus, at this point we avoid the routine use of antifibrinolytics to mitigate the coagulopathy in patients with APL. A potential avenue of investigation would be the use of additional coagulopathy testing such as TEG to determine the patients most likely to benefit from antifibrinolytics, however this approach requires clinical investigation before incorporation in standard care.

### Heparin

Heparin's ability to decrease intravascular fibrin formation and coagulation factor consumption due to disseminated intravascular coagulation has been the subject of much speculation and some investigation. Low doses of unfractionated heparin or low molecular weight heparin act on endothelial cells to protect them from unwanted interactions with malignant promyeloblasts (78). By interfering with excess clotting activation, low dose heparins (in the range of 5–10 units/kg/h) may decrease need of platelet and cryoprecipitate transfusions (79). Nevertheless, a retrospective study of 268 patients in the pre-ATRA era found no difference in early hemorrhagic death for those treated with prophylactic heparin compared with supportive care alone (76). In addition, more recent studies have raised the concern of increased hemorrhagic events, particularly delayed hemorrhage, with exposure to heparin (10). Thus, further research is necessary, and at this time, we do not recommend indiscriminate use of heparin in patients with APL.

Some centers have used recombinant factor VII for APL patients with an intracranial hemorrhage, but there is limited literature to support standard utilization in life-threatening hemorrhage at this time (80, 81). The use of recombinant thrombomodulin has been reported and was well-tolerated, though small patient populations limit the ability to judge clinical effectiveness (57, 59, 82).

Coagulopathy in APL shares some characteristics with DIC in sepsis, but the quantitative and qualitative platelet defects in APL are more profound (35). Agents such as antithrombin have shown no clear benefit in DIC, though the mechanism is an appealing target for study in APL-particularly in patients at highest risk for thrombotic events (83). Importantly, recombinant human activated protein C (APC) was removed from the marked after initial use for severe sepsis and DIC, in part due to risk of hemorrhage- a particularly concerning risk in patients with APL (84).

## Discussion

APL, more than any other acute leukemia, is marked by a characteristic coagulopathy at diagnosis. Hemorrhagic syndromes vary from easy bruising or purpura to hematomas and even intracranial hemorrhage, the most serious complication and the main cause of early death.

Since 1990, an immense body of basic science research has improved our understanding of the pathophysiology of coagulation in APL. Despite these advances in knowledge, interventional studies to prevent or manage hemorrhagic and thrombotic complications are infrequent and not powered to drive clinical practice forward. These failures to advance our management of coagulopathy in APL are underscored by the persistently high rates of early hemorrhagic and thrombotic death. While long-term outcomes have improved with the use of ATRA and ATO, the last major hurdle in APL is to prevent early hemorrhagic and thrombotic deaths.

Multiple questions face clinicians aiming to decrease hemorrhagic and thrombotic risk in APL. Are there markers which can reliably and reproducibly identify degree of endothelial damage in an individual patient? Can we identify patients more prone to hemorrhage vs. thrombosis, and tailor risk mitigation strategies based on individual coagulopathy profile? Are there any biomarkers or risk factors that would be particularly helpful in making these decisions? Moreover, as the risk for bleeding and clotting events is dynamic during induction therapy, how can we assess these changes in individual patients? The dynamic nature of coagulopathy in APL suggests that risk mitigation strategies should vary depending on time in therapy. Such an approach may transfuse platelets earlier in induction, shifting to more aggressive use of cryoprecipitate for a higher fibrinogen goal during the second week of therapy.

In addition to its role in APL, ATRA is able to differentiate non-APL myeloblasts. The relative contributions of annexin II, tissue factor, and cancer procoagulant to coagulopathy in non-APL acute leukemias are less well-understood, and further studies are needed to define if ATRA is able to mitigate the coagulopathy in other acute leukemias.

Coagulation remains profoundly altered despite the administration of ATRA, even if much improved in comparison with the pre-ATRA era. Interventions such as recombinant human soluble thrombomodulin to mitigate the risk of coagulopathy remain understudied. Moreover, the role of recombinant factor VII in life-threatening hemorrhage requires additional research. Likewise, the approach to anticoagulation after confirmed thrombosis remains a clinical challenge with little data to guide bedside clinicians. To adequately study the aforementioned interventions, many centers will have to collaborate prospectively and/or pool their retrospective experience in this relatively rare malignancy.

Such an effort will pose significant logistical challenges, but may finally leverage our pathophysiologic understanding into improved outcomes for patients with APL.

## Author Contributions

All authors contributed to the design, writing, and critical review of the manuscript.

## Conflict of Interest

The authors declare that the research was conducted in the absence of any commercial or financial relationships that could be construed as a potential conflict of interest.

## Publisher's Note

All claims expressed in this article are solely those of the authors and do not necessarily represent those of their affiliated organizations, or those of the publisher, the editors and the reviewers. Any product that may be evaluated in this article, or claim that may be made by its manufacturer, is not guaranteed or endorsed by the publisher.

## References

[1] Golomb HM Vardiman J Rowley JD. Acute nonlymphocytic leukemia in adults: correlations with Q-banded chromosomes. Blood. (1976) 48:9–21. 10.1182/blood.V48.1.9.9 59614

[2] Arber DA Orazi A Hasserjian R Thiele J Borowitz MJ Le Beau MM. The 2016 revision to the World Health Organization classification of myeloid neoplasms and acute leukemia. Blood. (2016) 127:2391–405. 10.1182/blood-2016-03-643544 27069254

[3] Tallman MS Andersen JW Schiffer CA Appelbaum FR Feusner JH Ogden A. All-trans-retinoic acid in acute promyelocytic leukemia. N Engl J Med. (1997) 337:1021–8. 10.1056/NEJM199710093371501 9321529

[4] Lo-Coco F Avvisati G Vignetti M Thiede C Orlando SM Iacobelli S. Retinoic acid and arsenic trioxide for acute promyelocytic leukemia. N Engl J Med. (2013) 369:111–21. 10.1056/NEJMoa1300874 23841729

[5] Iland HJ Bradstock K Supple SG Catalano A Collins M Hertzberg M. All-trans-retinoic acid, idarubicin, and IV arsenic trioxide as initial therapy in acute promyelocytic leukemia (APML4). Blood. (2012) 120:1570. 10.1182/blood-2012-02-410746 22715121

[6] Zhu H Hu J Chen L Zhou W Li X Wang L. The 12-year follow-up of survival, chronic adverse effects, and retention of arsenic in patients with acute promyelocytic leukemia. Blood. (2016) 128:1525–8. 10.1182/blood-2016-02-699439 27402972

[7] Park JH Qiao B Panageas KS Schymura MJ Jurcic JG Rosenblat TL. Early death rate in acute promyelocytic leukemia remains high despite all-trans retinoic acid. Blood. (2011) 118:1248–54. 10.1182/blood-2011-04-346437 21653939 PMC3790946

[8] Lehmann S Deneberg S Antunovic P Rangert-Derolf Å Garelius H Lazarevic V. Early death rates remain high in high-risk APL: update from the Swedish Acute Leukemia Registry 1997-2013. Leukemia. (2017) 31:1457–9. 10.1038/leu.2017.71 28232742

[9] Xu F Wang C Yin C Jiang X Jiang L Wang Z. Analysis of early death in newly diagnosed acute promyelocytic leukemia patients. Medicine (Baltimore). (2017) 96:e9324. 10.1097/MD.0000000000009324 29390508 PMC5758210

[10] Hambley BC Norsworthy KJ Jasem J Zimmerman JW Shenderov E Webster JA. Fibrinogen consumption and use of heparin are risk factors for delayed bleeding during acute promyelocytic leukemia induction. Leuk Res. (2019) 83:106174. 10.1016/j.leukres.2019.106174 31255938

[11] Mantha S Goldman DA Devlin SM Lee J-W Zannino D Collins M. Determinants of fatal bleeding during induction therapy for acute promyelocytic leukemia in the ATRA era. Blood. (2017) 129:1763–7. 10.1182/blood-2016-10-747170 28082441 PMC5374291

[12] Yanada M Matsushita T Asou N Kishimoto Y Tsuzuki M Maeda Y. Severe hemorrhagic complications during remission induction therapy for acute promyelocytic leukemia: incidence, risk factors, and influence on outcome. Eur J Haematol. (2007) 78:213–9. 10.1111/j.1600-0609.2006.00803.x 17241371

[13] Breccia M Avvisati G Latagliata R Carmosino I Guarini A De Propris MS. Occurrence of thrombotic events in acute promyelocytic leukemia correlates with consistent immunophenotypic and molecular features. Leukemia. (2007) 21:79–83. 10.1038/sj.leu.2404377 16932337

[14] Rashidi A Silverberg ML Conkling PR Fisher SI. Thrombosis in acute promyelocytic leukemia. Thromb Res. (2013) 131:281–9. 10.1016/j.thromres.2012.11.024 23266518

[15] Mitrovic M Suvajdzic N Elezovic I Bogdanovic A Djordjevic V Miljic P. Thrombotic events in acute promyelocytic leukemia. Thromb Res. (2015) 135:588–93. 10.1016/j.thromres.2014.11.026 25528069

[16] Chu Y Guo H Zhang Y Qiao R. Procoagulant platelets: generation, characteristics, and therapeutic target. J Clin Lab Anal. (2021) 35:e23750. 10.1002/jcla.23750 33709517 PMC8128296

[17] Sturn DH Kaneider NC Feistritzer C Djanani A Fukudome K Wiedermann CJ. Expression and function of the endothelial protein C receptor in human neutrophils. Blood. (2003) 102:1499–505. 10.1182/blood-2002-12-3880 12714492

[18] Mayer SA Brun NC Begtrup K Broderick J Davis S Diringer MN. Recombinant activated factor VII for acute intracerebral hemorrhage. New Eng J Med. (2005) 352:777–85. 10.1056/NEJMoa042991 15728810

[19] Garcia D Libby E Crowther MA. The new oral anticoagulants. Blood. (2010) 115:15–20. 10.1182/blood-2009-09-241851 19880491

[20] Cai J Ribkoff J Olson S Raghunathan V Al-Samkari H DeLoughery TG. The many roles of tranexamic acid: an overview of the clinical indications for TXA in medical and surgical patients. Eur J Haematol. (2020) 104:79–87. 10.1111/ejh.13348 31729076 PMC7023891

[21] Lee H-J Park H-J Kim H-W Park S-G. Comparison of laboratory characteristics between acute promyelocytic leukemia and other subtypes of acute myeloid leukemia with disseminated intravascular coagulation. Blood Res. (2013) 48:250–3. 10.5045/br.2013.48.4.250 24466548 PMC3894382

[22] Bennett B Booth NA Croll A Dawson AA. The bleeding disorder in acute promyelocytic leukaemia: fibrinolysis due to u-PA rather than defibrination. Br J Haematol. (1989) 71:511–7. 10.1111/j.1365-2141.1989.tb06311.x 2496742

[23] Menell JS Cesarman GM Jacovina AT McLaughlin MA Lev EA Hajjar KA. Annexin II and bleeding in acute promyelocytic leukemia. N Engl J Med. (1999) 340:994–1004. 10.1056/NEJM199904013401303 10099141

[24] Mantha S Tallman MS Soff GA. What's new in the pathogenesis of the coagulopathy in acute promyelocytic leukemia? Curr Opin Hematol. (2016) 23:121–6. 10.1097/MOH.0000000000000221 26760586

[25] Avvisati G ten Cate JW Sturk A Lamping R Petti MG Mandelli F. Acquired alpha-2-antiplasmin deficiency in acute promyelocytic leukaemia. Br J Haematol. (1988) 70:43–8. 10.1111/j.1365-2141.1988.tb02432.x 2460126

[26] Sakata Y Murakami T Noro A Mori K Matsuda M. The specific activity of plasminogen activator inhibitor-1 in disseminated intravascular coagulation with acute promyelocytic leukemia. Blood. (1991) 77:1949–57. 10.1182/blood.V77.9.1949.1949 1708294

[27] Wang P Zhang Y Yang H Hou W Jin B Hou J. Characteristics of fibrinolytic disorders in acute promyelocytic leukemia. Hematology. (2018) 23:756–64. 10.1080/10245332.2018.1470069 29724147

[28] Egbring R Schmidt W Fuchs G Havemann K. Demonstration of granulocytic proteases in plasma of patients with acute leukemia and septicemia with coagulation defects. Blood. (1977) 49:219–31. 10.1182/blood.V49.2.219.bloodjournal492219 299817

[29] Oudijk EJ Nieuwenhuis HK Bos R Fijnheer R. Elastase mediated fibrinolysis in acute promyelocytic leukemia. Thromb Haemost. (2000) 83:906–8. 10.1055/s-0037-1613942 10896247

[30] Yang R Zhong L Yang X-Q Jiang K-L Li L Song H. Neutrophil elastase enhances the proliferation and decreases apoptosis of leukemia cells via activation of PI3K/Akt signaling. Mol Med Rep. (2016) 13:4175–82. 10.3892/mmr.2016.5051 27035679 PMC4838072

[31] Yu L Zhong L Xiong L Dan W Li J Ye J. Neutrophil elastase-mediated proteolysis of the tumor suppressor p200 CUX1 promotes cell proliferation and inhibits cell differentiation in APL. Life Sci. (2020) 242:117229. 10.1016/j.lfs.2019.117229 31887298

[32] Bassi SC Rego EM. Tissue factor pathway inhibitor (TFPI) may be another important factor in the coagulopathy in acute promyelocytic leukemia (APL). Blood. (2015) 126:2278. 10.1182/blood.V126.23.2278.2278

[33] Kwaan HC Wang J Weiss I. Expression of receptors for plasminogen activators on endothelial cell surface depends on their origin. J Thromb Haemost. (2004) 2:306–12. 10.1111/j.1538-7933.2004.00593.x 14995994

[34] Song Y Peng P Qiao C Zhang R Li J Lu H. Low platelet count is potentially the most important contributor to severe bleeding in patients newly diagnosed with acute promyelocytic leukemia. Onco Targets Ther. (2017) 10:4917–24. 10.2147/OTT.S144438 29062237 PMC5640392

[35] Psaila B Bussel JB Frelinger AL Babula B Linden MD Li Y. Differences in platelet function in patients with acute myeloid leukemia and myelodysplasia compared to equally thrombocytopenic patients with immune thrombocytopenia. J Thromb Haemost. (2011) 9:2302–10. 10.1111/j.1538-7836.2011.04506.x 21920014 PMC3210015

[36] Asakura H Saito M Ito K Jokaji Y Uotani C Kumabashiri I. Levels of thrombin-antithrombin III complex in plasma in cases of acute promyelocytic leukemia. Thromb Res. (1988) 50:895–9. 10.1016/0049-3848(88)90349-0 3166241

[37] Chen C-Y Tai C-H Tsay W Chen P-Y Tien H-F. Prediction of fatal intracranial hemorrhage in patients with acute myeloid leukemia. Ann Oncol. (2009) 20:1100–4. 10.1093/annonc/mdn755 19270342

[38] Ho G Li Q Brunson A Jonas BA Wun T Keegan THM. Complications and early mortality in patients with acute promyelocytic leukemia treated in California. Am J Hematol. (2018) 93:E370–2. 10.1002/ajh.25252 30105792 PMC6486372

[39] de la Serna J Montesinos P Vellenga E Rayón C Parody R León A. Causes and prognostic factors of remission induction failure in patients with acute promyelocytic leukemia treated with all-trans retinoic acid and idarubicin. Blood. (2008) 111:3395–402. 10.1182/blood-2007-07-100669 18195095

[40] Asou N Kishimoto Y Kiyoi H Okada M Kawai Y Tsuzuki M. A randomized study with or without intensified maintenance chemotherapy in patients with acute promyelocytic leukemia who have become negative for PML-RARα transcript after consolidation therapy: the Japan Adult Leukemia Study Group (JALSG) APL97 study. Blood. (2007) 110:59–66. 10.1182/blood-2006-08-043992 17374742

[41] Dally N Hoffman R Haddad N Sarig G Rowe JM Brenner B. Predictive factors of bleeding and thrombosis during induction therapy in acute promyelocytic leukemia—a single center experience in 34 patients. Thrombosis Research. (2005) 116:109–14. 10.1016/j.thromres.2004.11.001 15907524

[42] Kim D-Y Lee J-H Lee J-H Kim S-D Lim S-N Choi Y. Significance of fibrinogen, D-dimer, and LDH levels in predicting the risk of bleeding in patients with acute promyelocytic leukemia. Leuk Res. (2011) 35:152–8. 10.1016/j.leukres.2010.05.022 20554322

[43] Mitrovic M Suvajdzic N Bogdanovic A Kurtovic NK Sretenovic A Elezovic I. International Society of Thrombosis and Hemostasis Scoring System for disseminated intravascular coagulation ≥ 6: a new predictor of hemorrhagic early death in acute promyelocytic leukemia. Med Oncol. (2013) 30:478. 10.1007/s12032-013-0478-y 23371042

[44] Minamiguchi H Fujita H Atsuta Y Asou N Sakura T Ueda Y. Predictors of early death, serious hemorrhage, and differentiation syndrome in Japanese patients with acute promyelocytic leukemia. Ann Hematol. (2020) 99:2787–800. 10.1007/s00277-020-04245-6 32879992

[45] Chang H Kuo M-C Shih L-Y Dunn P Wang P-N Wu J-H. Clinical bleeding events and laboratory coagulation profiles in acute promyelocytic leukemia. Eur J Haematol. (2012) 88:321–8. 10.1111/j.1600-0609.2011.01747.x 22221178

[46] Bao H Du J Chen B Wang Y. The role of thromboelastography in predicting hemorrhage risk in patients with leukemia. Medicine (Baltimore). (2018) 97:e0137. 10.1097/MD.0000000000010137 29595638 PMC5895378

[47] Ciftciler R Haznedaroglu IC Aksu S Ozcebe O Sayinalp N Malkan UY. The factors affecting early death in newly diagnosed APL patients. Open Med (Wars). (2019) 14:647–52. 10.1515/med-2019-0074 31565673 PMC6744608

[48] Ma G Liu F Lv L Gao Y Su Y. Increased promyelocytic-derived microparticles: a novel potential factor for coagulopathy in acute promyelocytic leukemia. Ann Hematol. (2013) 92:645–52. 10.1007/s00277-013-1676-6 23344645

[49] Kwaan HC Rego EM McMachon B Weiss I. Thrombin generationand fibrinolytic activity in microparticles in acute promyelocytic leukemia. Blood. (2013) 122:3620. 10.1182/blood.V122.21.3620.3620

[50] Langer F Spath B Haubold K Holstein K Marx G Wierecky J. Tissue factor procoagulant activity of plasma microparticles in patients with cancer-associated disseminated intravascular coagulation. Ann Hematol. (2008) 87:451–7. 10.1007/s00277-008-0446-3 18292996

[51] Falanga A Consonni R Marchetti M Locatelli G Garattini E Passerini CG. Cancer procoagulant and tissue factor are differently modulated by all-trans-retinoic acid in acute promyelocytic leukemia cells. Blood. (1998) 92:143–51. 10.1182/blood.V92.1.143.413k18_143_151 9639510

[52] Dubois C Schlageter MH de Gentile A Guidez F Balitrand N Toubert ME. Hematopoietic growth factor expression and ATRA sensitivity in acute promyelocytic blast cells. Blood. (1994) 83:3264–70. 10.1182/blood.V83.11.3264.bloodjournal83113264 8193361

[53] Schorer AE Kaplan ME Rao GH Moldow CF. Interleukin 1 stimulates endothelial cell tissue factor production and expression by a prostaglandin-independent mechanism. Thromb Haemost. (1986) 56:256–9. 10.1055/s-0038-1661661 3031841

[54] Schleef RR Bevilacqua MP Sawdey M Gimbrone MA Loskutoff DJ. Cytokine activation of vascular endothelium. Effects on tissue-type plasminogen activator and type 1 plasminogen activator inhibitor. J Biol Chem. (1988) 263:5797–803. 10.1016/S0021-9258(18)60636-2 3128548

[55] Lentz SR Tsiang M Sadler JE. Regulation of thrombomodulin by tumor necrosis factor-alpha: comparison of transcriptional and posttranscriptional mechanisms. Blood. (1991) 77:542–50. 10.1182/blood.V77.3.542.bloodjournal773542 1846763

[56] Nan B Lin P Lumsden AB Yao Q Chen C. Effects of TNF-alpha and curcumin on the expression of thrombomodulin and endothelial protein C receptor in human endothelial cells. Thromb Res. (2005) 115:417–26. 10.1016/j.thromres.2004.10.010 15733976

[57] Ikezoe T Takeuchi A Isaka M Arakawa Y Iwabu N Kin T. Recombinant human soluble thrombomodulin safely and effectively rescues acute promyelocytic leukemia patients from disseminated intravascular coagulation. Leuk Res. (2012) 36:1398–402. 10.1016/j.leukres.2012.08.012 22917769

[58] Ikezoe T. Pathogenesis of disseminated intravascular coagulation in patients with acute promyelocytic leukemia, and its treatment using recombinant human soluble thrombomodulin. Int J Hematol. (2014) 100:27–37. 10.1007/s12185-013-1463-0 24217998

[59] Matsuda K Jo T Toyama K Nakazaki K Matsui H Fushimi K. Efficacy of recombinant human soluble thrombomodulin in induction therapy for acute promyelocytic leukemia. Thromb Res. (2021) 202:173–5. 10.1016/j.thromres.2021.04.003 33866226

[60] Montesinos P de la Serna J Vellenga E Rayon C Bergua J Parody R. Incidence and risk factors for thrombosis in patients with acute promyelocytic leukemia. Experience of the PETHEMA LPA96 and LPA99 protocols. Blood. (2006) 108:1503. 10.1182/blood.V108.11.1503.1503

[61] Escudier SM Kantarjian HM Estey EH. Thrombosis in patients with acute promyelocytic leukemia treated with and without all-trans retinoic acid. Leuk Lymphoma. (1996) 20:435–9. 10.3109/10428199609052425 8833399

[62] Chang H Kuo M-C Shih L-Y Wu J-H Lin T-L Dunn P. Acute promyelocytic leukemia-associated thrombosis. Acta Haematol. (2013) 130:1–6. 10.1159/000345833 23343825

[63] Bai Y Shi M Yang X Zhang W Yang R Wei X. The value of FDP/FIB and D-dimer/FIB ratios in predicting high-risk APL-related thrombosis. Leuk Res. (2019) 79:34–7. 10.1016/j.leukres.2019.02.007 30831481

[64] Sanz MA Fenaux P Tallman MS Estey EH Lowenberg B Naoe T. Management of acute promyelocytic leukemia: updated recommendations from an expert panel of the European LeukemiaNet. Blood. (2019) 133:1630–43. 10.1182/blood-2019-01-894980 30803991 PMC6509567

[65] Barbui T Finazzi G Falanga A. The impact of all-trans-retinoic acid on the coagulopathy of acute promyelocytic leukemia. Blood. (1998) 91:3093–102. 10.1182/blood.V91.9.3093 9558362

[66] Burnett AK Russell NH Hills RK Bowen D Kell J Knapper S. Arsenic trioxide and all-trans retinoic acid treatment for acute promyelocytic leukaemia in all risk groups (AML17): results of a randomised, controlled, phase 3 trial. Lancet Oncol. (2015) 16:1295–305. 10.1016/S1470-2045(15)00193-X 26384238

[67] Azuma Y Nakaya A Hotta M Fujita S Tsubokura Y Yoshimura H. Disseminated intravascular coagulation observed following treatment with gemtuzumab ozogamicin for relapsed/refractory acute promyelocytic leukemia. Mol Clin Oncol. (2016) 5:31–4. 10.3892/mco.2016.864 27330760 PMC4906953

[68] Tallman MS Lefèbvre P Baine RM Shoji M Cohen I Green D. Effects of all-trans retinoic acid or chemotherapy on the molecular regulation of systemic blood coagulation and fibrinolysis in patients with acute promyelocytic leukemia. J Thromb Haemost. (2004) 2:1341–50. 10.1111/j.1538-7836.2004.00787.x 15304040

[69] Olwill SA McGlynn H Gilmore WS Alexander HD. All-trans retinoic acid-induced downregulation of annexin II expression in myeloid leukaemia cell lines is not confined to acute promyelocytic leukaemia. Br J Haematol. (2005) 131:258–64. 10.1111/j.1365-2141.2005.05750.x 16197459

[70] Koyama T Hirosawa S Kawamata N Tohda S Aoki N. All-trans retinoic acid upregulates thrombomodulin and downregulates tissue-factor expression in acute promyelocytic leukemia cells: distinct expression of thrombomodulin and tissue factor in human leukemic cells. Blood. (1994) 84:3001–9. 10.1182/blood.V84.9.3001.bloodjournal8493001 7949172

[71] De Stefano V Teofili L Sica S Mastrangelo S Di Mario A Rutella S. Effect of all-trans retinoic acid on procoagulant and fibrinolytic activities of cultured blast cells from patients with acute promyelocytic leukemia. Blood. (1995) 86:3535–41. 10.1182/blood.V86.9.3535.bloodjournal8693535 7579461

[72] Wartha F Henriques-Normark B. ETosis: a novel cell death pathway. Sci Signal. (2008) 1:pe25. 10.1126/stke.121pe25 18506034

[73] Swystun LL Liaw PC. The role of leukocytes in thrombosis. Blood. (2016) 128:753–62. 10.1182/blood-2016-05-718114 27354721

[74] Fuchs TA Brill A Duerschmied D Schatzberg D Monestier M Myers DD. Extracellular DNA traps promote thrombosis. Proc Natl Acad Sci USA. (2010) 107:15880–5. 10.1073/pnas.1005743107 20798043 PMC2936604

[75] Fuchs TA Brill A Wagner DD. Neutrophil extracellular trap (NET) impact on deep vein thrombosis. Arterioscler Thromb Vasc Biol. (2012) 32:1777–83. 10.1161/ATVBAHA.111.242859 22652600 PMC3495595

[76] Rodeghiero F Avvisati G Castaman G Barbui T Mandelli F. Early deaths and anti-hemorrhagic treatments in acute promyelocytic leukemia. A GIMEMA retrospective study in 268 consecutive patients. Blood. (1990) 75:2112–7. 10.1182/blood.V75.11.2112.bloodjournal75112112 2189506

[77] Sanz MA Montesinos P. Open issues on bleeding and thrombosis in acute promyelocytic leukemia. Thromb Res. (2010) 125:S51–4. 10.1016/S0049-3848(10)70013-X 20434005

[78] Vignoli A Marchetti M Falanga A. Acute promyelocytic leukemia cell adhesion to vascular endothelium is reduced by heparins. Ann Hematol. (2018) 97:1555–62. 10.1007/s00277-018-3343-4 29717364

[79] Liu X-L Wang X-Z Liu X-X Hao D Jaladat Y Lu F. Low-dose heparin as treatment for early disseminated intravascular coagulation during sepsis: a prospective clinical study. Exp Ther Med. (2014) 7:604–8. 10.3892/etm.2013.1466 24520253 PMC3919907

[80] Zver S Andoljšek D Cernelč P. Effective treatment of life-threatening bleeding with recombinant activated factor VII in a patient with acute promyelocytic leukaemia. Eur J Haematol. (2004) 72:455–6. 10.1111/j.1600-0609.2004.00237.x 15128428

[81] Nosari A Caimi TM Zilioli V Molteni A Mancini V Morra E. Cerebral hemorrhage treated with NovoSeven in acute promyelocytic leukemia. Leuk Lymphoma. (2012) 53:160–1. 10.3109/10428194.2011.605189 21780994

[82] Ookura M Hosono N Tasaki T Oiwa K Fujita K Ito K. Successful treatment of disseminated intravascular coagulation by recombinant human soluble thrombomodulin in patients with acute myeloid leukemia. Medicine (Baltimore). (2018) 97:e12981. 10.1097/MD.0000000000012981 30383650 PMC6221668

[83] Kim Y-J Ko BS Park SY Oh DK Hong S-B Jang S. Effect of high-dose antithrombin supplementation in patients with septic shock and disseminated intravascular coagulation. Sci Rep. (2019) 9:16626. 10.1038/s41598-019-52968-y 31719571 PMC6851090

[84] Ikezoe T. Thrombomodulin/activated protein C system in septic disseminated intravascular coagulation. J Intensive Care. (2015) 3:1. 10.1186/s40560-014-0050-7 25705426 PMC4336127

